# How Size Matters: Diversity for Fragment Library Design

**DOI:** 10.3390/molecules24152838

**Published:** 2019-08-05

**Authors:** Yun Shi, Mark von Itzstein

**Affiliations:** Institute for Glycomics, Griffith University, Gold Coast Campus, Gold Coast, Queensland 4222, Australia

**Keywords:** diversity, fragment-based drug discovery, library design, library size

## Abstract

Fragment-based drug discovery (FBDD) has become a major strategy to derive novel lead candidates for various therapeutic targets, as it promises efficient exploration of chemical space by employing fragment-sized (MW < 300) compounds. One of the first challenges in implementing a FBDD approach is the design of a fragment library, and more specifically, the choice of its size and individual members. A diverse set of fragments is required to maximize the chances of discovering novel hit compounds. However, the exact diversity of a certain collection of fragments remains underdefined, which hinders direct comparisons among different selections of fragments. Based on structural fingerprints, we herein introduced quantitative metrics for the structural diversity of fragment libraries. Structures of commercially available fragments were retrieved from the ZINC database, from which libraries with sizes ranging from 100 to 100,000 compounds were selected. The selected libraries were evaluated and compared quantitatively, resulting in interesting size-diversity relationships. Our results demonstrated that while library size does matter for its diversity, there exists an optimal size for structural diversity. It is also suggested that such quantitative measures can guide the design of diverse fragment libraries under different circumstances.

## 1. Introduction

Fragment-based drug discovery (FBDD) has been developed in the past twenty years as an approach to derive novel lead compounds for various therapeutic targets [1,2,3,4]. It features the use of fragment-sized compounds that mostly comply with the ‘Rule-of-3′ [5] for the identification of hits, which can be subsequently developed into potent lead compounds. Compared to the more traditional high-throughput screening that employs drug-like compounds following the ‘Rule-of-5′ [6], the smaller sizes of fragments used in FBDD lead to more efficient sampling of the relevant chemical space and thus better chances of identifying novel hits [7]. The smaller sizes also result in higher ligand efficiency [8] and more efficient structural optimization of fragment hits [9]. With these advantages, FBDD has gained popularity in both academia and industry in recent years [10], and led to the discovery of three Food and Drug Administration-approved drugs [11,12,13,14].

The first and foremost step in FBDD is the design of a fragment library, as library compositions directly influence the outcome of FBDD projects. One of the most frequently discussed topics for its design is the size of the fragment library, which has a substantial impact on the early stages as it affects the time and monetary costs in addition to the outcome of FBDD projects. Interestingly, the majority of respondents in recent polls had up to 2,000 compounds in their fragment libraries [15,16], while recent successful FBDD campaigns had library sizes of between 1,000 and 2,000 compounds [17,18]. Besides an optimal library size, consideration is also given to the structural complexity, physicochemical profile, and shape profile of fragments [19]. However, the *diversity* of a fragment library should be the most critical factor, because it affects the sampling efficiency of the relevant chemical space as well as the novelty of potential hit compounds. Of note, better diversity should decrease the screening hit rate, which appears to be high for many FBDD campaigns [4,20]. Hence, the size of a fragment library should be discussed in conjunction with its diversity.

Diversity needs to be characterized by descriptors, which can be classified mostly into three categories. The first are functional (performance) descriptors based on the bioactivities of compounds towards a panel of (functionally dissimilar) biological targets [21]. Although regarded as the most relevant category of diversity descriptors for drug discovery [22,23], acquisition of bioactivity data can be very resource-demanding [24,25]. In addition to a lack of bioactivity data for fragment-sized compounds in the literature, their activities would also be difficult to detect and measure due to their weak affinities [7]. The second are physicochemical (property-based) descriptors, including common physicochemical properties such as molecular weight, hydrophobicity, and even electronic properties [26]. The third are structural descriptors, among which molecular fingerprints (structural features) are routinely used to represent chemical structures. The extended-connectivity (radial) fingerprints [27] is effective at retrieving bioactive compounds [28], therefore it was chosen as the descriptor of diversity in our study.

There are currently two major types of quantitative metrics for structural diversity [29]. The first type of metrics assesses the similarity (and thus difference) between pairs of chemical structures. The most notable metric of this type is the Jaccard index [30], later referred to as the popular Tanimoto index (similarity) [31]. The second type of metrics calculates the coverage of the relevant chemical space by a library of compounds, and the most straightforward one is a ratio based on richness, defined as the number of unique fingerprints (structural features) [32]. In this work, we propose the adoption of a third type of metrics, i.e., a diversity index that takes into account not only the number of unique structural fingerprints but also their proportional abundances [33,34,35], for the quantitative measurement of diversity. True diversity, or the effective number of structural features, is a commonly used metric of this type and can be defined by the following Equation (1) [35]:

(1) $$D=\frac{1}{\prod_{i=1}^{R}p_{i}^{p_{i}}}$$

where D stands for true diversity, R is richness (the total number of fingerprints), and $p_{i}$ represents the proportional abundance of the ith fingerprint. It can be deduced from Equation (1) that, for the same richness, a library with a more even distribution of proportional abundances will have a larger true diversity than a library with a less even distribution. These diversity indexes have been used in ecological studies for decades, yet they have not been applied to the measurement of diversity of fragment libraries to date. Although there are other plot-based methods to illustrate diversity in more visually appealing ways, such as principle component analysis [36] and principal moments of inertia [37,38], these three quantitative metrics, i.e., Tanimoto similarity, number of fingerprints, and true diversity, are more suited for direct comparison of libraries with different sizes.

To provide insights into how the library size affects the structural diversity, we herein compare fragment libraries of different sizes, selected from commercially available fragments, and demonstrate interesting size-diversity relationships. Such relationships indicated the presence of an optimal library size for structural diversity. We also extend this investigation to a more restrictive scenario, in which only fluorinated fragments are considered and consequentially similar size-diversity relationships were observed. Certain cost-effective sizes that capture significant proportions of the overall diversity available with very small portions of available fragments are also proposed. Our results demonstrated that these quantitative metrics could assist in the design of fragment libraries under various circumstances.

## 2. Results

### 2.1. Library Selection

To generate libraries for comparison, both diversity-based selections and random selections were performed from 227,787 commercially available fragments that had undergone filtering by the ‘Rule-of-3′ criteria [5]. Libraries with sizes of 100, 200, 500, 1,000, 2,000, 5,000, 10,000, 20,000, 50,000, and 100,000 were selected. Both diversity-based selections and random selections were performed, with the latter in triplicate. To demonstrate that our approach can be applied to different circumstances, selections were also performed on a fluorinated subset of the 227,787 commercially available fragments, consisting of 47,708 fragments that has 1~3 fluorine atoms. Such restriction on the number of fluorine atoms captured the majority of fluorinated fragments, which are commonly used for FBDD projects employing ^19^F NMR as the screening method [39,40]. Fluorinated libraries with sizes of 100, 200, 500, 1,000, 2,000, 5,000, 10,000, and 20,000 were selected in similar fashions.

### 2.2. Size-Diversity Relationship of Regular Fragment Libraries

**Figure 1:**
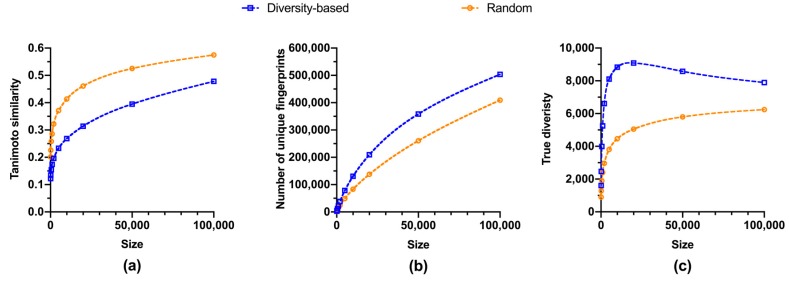
Structural diversity vs size of fragment libraries, with the former measured by: (**a**) Average of the similarity of each compound to its closest neighbor; (**b**) total number of unique fingerprints (richness); (**c**) true diversity calculated by equation 1. Dash curves are generated from cubic spline fitting. Metrics for random selections are average values of triplicates (Table S2).

**Figure 2:**
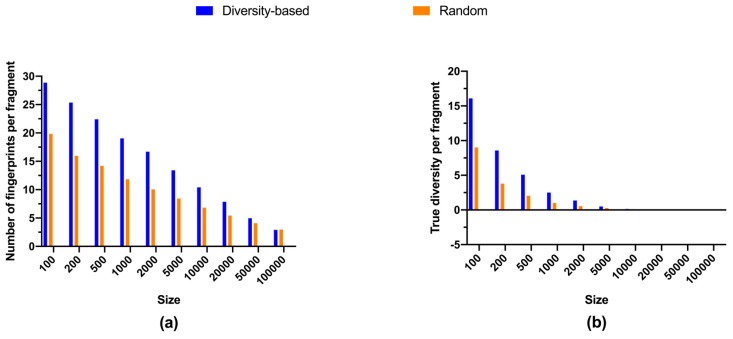
Efficiency in adding diversity: (**a**) average number of unique fingerprints (richness) per compound; (**b**) average value of true diversity per compound. Metrics for random selections are average values of triplicates.

To understand the relationship between the size of fragment libraries and their structural diversity, quantitative metrics were calculated for selected libraries (Figure 1). As expected, fragments became more similar to each other as the library size increased, and the diversity-based selection did lead to more dissimilar fragments than random selections (Figure 1a). Richness of fragment library also rose with its size, with diversity-based selections outperforming random selections (Figure 1b). However, marginal richness, i.e., the additional number of unique fingerprints per additional fragment, was declining while library size grew (Figure 2a). For diversity-based selections, the average efficiency of adding unique fingerprints from 2,000 fragments to 5,000 fragments, 13.4 fingerprints per compound, was less than half of that from nothing to 100 fragments, 28.9 fingerprints per compound. Similar trends were observed for randomly selected libraries, although the gap between diversity-based and random selections became smaller when library sizes grew excessively large, i.e., beyond 5,000 compounds. Thus, it is more efficient to have relatively small library for richness and we estimated the number of fragments required to accomplish two arbitrary degrees of coverage, 5% and 10%, respectively (Table 1). These two cut-offs are convenient numbers chosen to manifest the coverage efficiency of small libraries.

Surprisingly, values of true diversity exhibited different trends between diversity-based selections and random selections (Figure 1c). While the latter showed a constantly rising movement, the former reached a maximum at about 18,000 fragments, representing less than 8% of the overall available fragments (Table 1), before starting to decline (Figure 1c). In addition, marginal true diversity experienced a more drastic decline in comparison with the marginal richness (Figure 2). For diversity-based selections, the average efficiency of adding true diversity from 2,000 fragments to 5,000 fragments, 1.4 per compound, was an order of magnitude less that from nothing to 100 fragments, 16.1 per compound. Consistent with the decline of true diversity after the library size from diversity-based selections reached about 18,000, the marginal true diversity became negative after 20,000 compounds (Figure 2b). More strikingly, only approximately 2,000 fragments, i.e., less than 1%, are required to attain the same level of true diversity as all of the 227,787 fragments available for selection (Table 1).

### 2.3. Size-Diversity Relationship of Fluorinated Fragment Libraries

**Figure 3:**
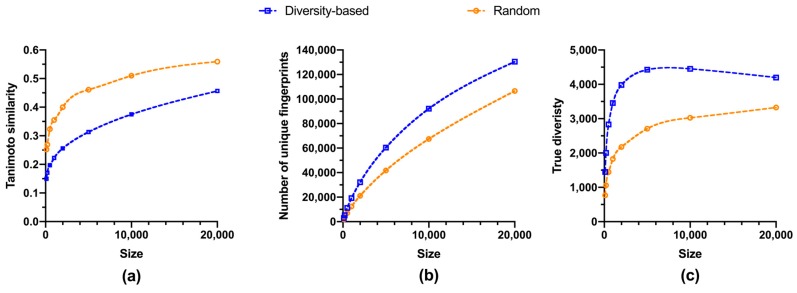
Structural diversity vs size of fluorinated fragment libraries, with the former measured by: (**a**) Average of the similarity of each compound to its closest neighbor; (**b**) total number of unique fingerprints (richness); (**c**) true diversity calculated by equation 1. Dash curves are generated from cubic spline fitting. Metrics for random selections are average values of triplicates (Table S2).

**Figure 4:**
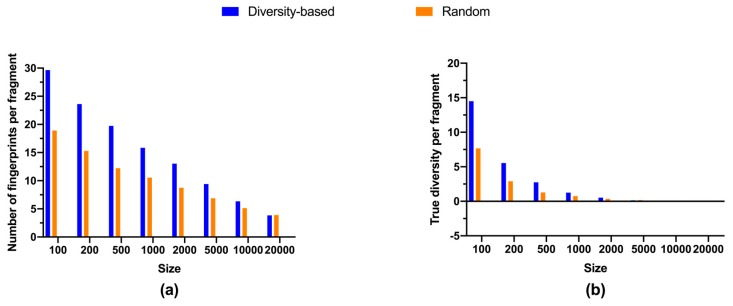
Efficiency in adding diversity: (**a**) average number of unique fingerprints (richness) per fluorinated compound; (**b**) average value of true diversity per fluorinated compound. Metrics for random selections are average values of triplicates.

Libraries selected from fluorinated fragments presented similar size-diversity relationships as those from regular fragments (Figure 3 and Figure 4, Table 2). Both similarity to the closest neighbor and richness illustrated growing trends (Figure 3a,b), whereas the true diversity for libraries subject to diversity-based selection also reached a maximum at about 7,500 fragments (Figure 3c and Table 2). Analogously, both marginal richness and marginal true diversity diminished with increasing library size, while the gap between diversity-based and random selections in efficiency became smaller for larger library sizes, i.e., beyond 500 compounds (Figure 4). Nevertheless, it required relatively more fluorinated fragments to achieve the same level of diversity than that for regular fragments. About 3.4% of total fluorinated fragments were needed to attain 10% coverage (Table 2), much higher than that for regular fragments, about 1.8%. Additionally, it took close to 15.7% of total fluorinated fragments to reach maximum true diversity, while for regular fragments only about 7.8% were required. Further, 2.5% of total fluorinated fragments were required to achieve the same level of true diversity as all the 47,708 fluorinated fragments, whereas less than 1% of regular fragments were required. These observations can be explained by the constant presence of fluorine atoms, and thus fluorine-containing fingerprints, in all fluorinated compounds. Inevitably, there would be a larger overlap of fluorine-associated fingerprints among fluorinated compounds, rendering the distribution of proportional abundances for fingerprints less even and thereby a smaller value of true diversity calculated by equation 1. Such a phenomenon can also be expected for other restrictive circumstances demanding the presence of certain functional groups and/or pharmacophores.

## 3. Discussion

The exact size-diversity relationships for fragment libraries are affected by several factors, including the fragments available for selection, the selection method, and the diversity metric. Using fluorinated fragments as an example, we have shown that similar size-diversity relationships are observed for this subset of available fragments. Thus, we speculate that different but similar size-diversity relationships could be observed for a different set of fragments available for selection. This could be either more restrictive, such as a set of fragments from a certain vendor, or more inclusive, such as a virtual set of all theoretically possible fragments [41]. Moreover, we expect that a different selection method, such as a clustering method [42], would offer somewhat different results (Table S1). Yet it should be noted that clustering methods are much less efficient than the directed sphere exclusion method used in this study [43], which features good computational performance on large data sets and enabled our calculations to be carried out on a desktop computer. Furthermore, our results illustrated that different diversity metrics could indeed show very different size-diversity relationships. While both similarity and richness increased with the size of fragment library, the rate of increase experienced a more significant decline in the former than in the latter, resulting in larger curvatures of the fitted lines for similarity. In contrast, the true diversity of libraries from diversity-based selections started to decrease after a certain size, highlighting the uneven distribution of structural fingerprints as the library size grew excessively large.

Not unexpectedly, our results showed that the marginal diversity diminishes while the library size increases, the extent and significance of which depends on the choice of diversity metrics. This indicates that it is unnecessary and possibly counterproductive to play numbers game and build excessively large libraries, and that cost-effective sizes of fragment library exist for structural diversity. For regular fragments selected from commercially readily available compounds, we propose a library size of ~2,000 (File S2), corresponding to 0.9% of total available fragments in this study. This size covers more than 5% of richness, approximates the true diversity of all available fragments, and (perhaps coincidentally) matches the most popular fragment library size [15,16]. For the fluorinated subset, a library size of ~1,200 (File S3) achieves similar coverage of richness and true diversity. However, better selection methods may even reduce these proposed numbers.

In addition to structural diversity, considerations should also be given to practical factors such as experimental solubility, (absence of) aggregation, and stability for fragment library design [19]. These factors are essential for the success of FBDD campaigns, yet they are difficult to predict without experimental data. Hence, it would be more pragmatic to slightly increase the library size in the initial *in silico* design and perform necessary quality checks after procurement of fragments.

In summary, we have introduced quantitative metrics to evaluate the structural diversity of fragment libraries, investigated their size-diversity relationships, and demonstrated the existence of an optimal library size for structural diversity depending on specific situations. Based on our results, we propose the use of relatively small library sizes and the application of these quantitative measures to the design of diverse fragment libraries under various circumstances.

## 4. Materials and Methods

Structures of commercially-available, fragment-sized compounds were retrieved from the ZINC 15 database [44] (https://zinc15.docking.org/tranches/home/) in SMILES format on 2 Jan 2019. A subset was chosen with the following criteria: Anodyne for Reactivity; In-Stock for Purchasability; up to 300 Daltons for Molecular Weight; up to 3 for LogP. These criteria resulted in 1,413,973 compounds. The Canvas program (Schrödinger, LLC, New York, NY, USA) was used for subsequent calculations. Physicochemical properties were calculated by canvasMolDescriptors and compounds violating an adapted version of the ‘Rule-of-3′ [5], i.e., 100 ≤ MW ≤ 300, logP ≤ 3, number of rings ≤ 3, number of hydrogen bond donors (HBD) ≤ 3, number of hydrogen bond acceptors (HBA) ≤ 3, number of rotatable bonds (RB) ≤ 3, and polar surface area ≤ 60 Å^2^ were removed. HBD, HBA, and RB are custom defined according to a previous work [45]. Any compound with reactive groups was filtered by the ligfilter functionality and duplicate structures were eliminated by the uniquesmiles functionality. Finally, 227,787 compounds (File S1 and Figure S1) were left for selection of fragment libraries.

Radial fingerprints [27] were generated by canvasFPGen, with 64-bit precision (2^64^) to avoid fingerprint collisions, Daylight invariant atom types [46], and three radial iterations. Based on the these fingerprints, diversity-based selections were performed with canvasDBCS, using the directed sphere exclusion method [43] and Tanimoto similarity [31]. An exclusion sphere size of 0.4 was used to select libraries with a maximum size of 100,000 compounds. In parallel, random selections of fragment libraries as control were carried out in triplicate by the UNIX command *shuf*. To quantify the diversity of selected libraries, three different metrics were calculated as follows: maximum Tanimoto similarity [31] was computed by canvasFPHist; total number of unique fingerprints [32] was counted by canvasFPBinary2CSV; and true diversity [35] was determined by the UNIX command *awk* using Equation (1).

For fluorinated fragments, the ligfilter functionality was used to filter the 227,787 compounds with a criterion of 1 ≤ number of fluorine atoms ≤ 3, and the resulting 47,708 fragments were subject to analogous calculations and selections with a maximum library size of 20,000 compounds.

Prism 8 (GraphPad Software, Inc., La Jolla, CA, USA) was employed to generate plots of the aforementioned three metrics against the size of selected libraries, and the cubic spline function was used to fit spine curves.

## Figures and Tables

**Table 1:** Library sizes (diversity-based selection) required to achieve certain values of structural diversity.

Structural Diversity (Value)	Minimum Size (Ratio of Total 227,787 Fragments) ^1^
5% total richness ^2^ (33,834)	1,715 (0.75%)
10% total richness ^2^ (67,669)	4,103 (1.80%)
Overall true diversity (6,662.4)	2,052 (0.90%)
Maximum true diversity ^1^ (9,097.6)	17,666 (7.76%)

^1^ Values are estimated by cubic spline fitting with 99,901 segments; ^2^ Total richness (number of unique fingerprints) is 676,686.

**Table 2:** Fluorinated library sizes (diversity-based selection) required to achieve certain values of structural diversity.

Structural Diversity (Value)	Minimum Size (Ratio of Total 47,708 Fluorinated Fragments) ^1^
5% total richness ^2^ (8,992)	675 (1.41%)
10% total richness ^2^ (17,983)	1,616 (3.39%)
Overall true diversity (3,621.9)	1,203 (2.52%)
Maximum true diversity ^1^ (4,485.5)	7,483 (15.69%)

^1^ Values are estimated by cubic spline fitting with 19,901 segments; ^2^ Total richness (number of unique fingerprints) is 179,833.

## Supplementary Materials

The following are available online at https://www.mdpi.com/1420-3049/24/15/2838/s1, File S1: List of SMILES structures of all 227,787 fragments used in this study; File S2: List of SMILES structures of 2,000 regular fragments proposed for cost-effectiveness; File S3: List of SMILES structures of 1,200 fluorinated fragments proposed for cost-effectiveness; Table S1: Comparison of clustering-based selections and diversity-base selections from a random set of 10,000 fragments; Table S2: Numerical values of diversity metrics calculated for all selected libraries; Figure S1: Chemical structures of 40 example fragments randomly selected from the 227,787 compounds used for library selections.
