# Supplementary material for: How Size Matters: Diversity for Fragment Library Design
Source: Molecules. 2019 Aug 5;24(15):2838. doi: 10.3390/molecules24152838 (PMC6696339; doi:10.3390/molecules24152838)
Supplement: Supplementary file 1 [file molecules-24-02838-s001.zip › Table S1.docx]

**Table S1.** Comparison of clustering-based selections and diversity-base selections from a random set of 10,000 fragments.

| **Selected size** | **Selection method** | **Compute time^1^ (sec)** | **Similarity^2^** | **Richness^2^** | **True diversity^2^** |
| --- | --- | --- | --- | --- | --- |
| 100 | Clustering-based^3^ | 42.22 | 0.247 | 1,355 | 605.25 |
| 100 | Diversity-based^4^ | 17.48 | 0.128 | 2,881 | 1,588.12 |
| 500 | Clustering-based^3^ | 182.47 | 0.286 | 6,592 | 1,598.00 |
| 500 | Diversity-based^4^ | 17.39 | 0.166 | 11,273 | 3,342.24 |

^1^ Computation for compound selections were performed on a Desktop computer (iMac equipped with Intel Core i7 4.2 GHz) using a single processor.

^2^ There were measured with the same metrics as in the main text.

^3^ Clustering-based selections were implemented by canvasKMeans of Canvas (Schrödinger, LLC), with commands: “$SCHRODINGER/utilities/canvasKMeans -JOB cl100_from_10k -ifp rad64day3_R10k -k 100 -o cl100” and “$SCHRODINGER/utilities/canvasKMeans -JOB cl500_from_10k -ifp rad64day3_R10k -k 500 -o cl500”.

^4^ Diversity-based selections were performed as described in the main text, with commands: “$SCHRODINGER/utilities/canvasDBCS -JOB dise100_from_10k -ifp rad64day3_R10k -method dise -metric tanimoto -n 100 -d 0.4 -o dise100.out” and “$SCHRODINGER/utilities/canvasDBCS -JOB dise500_from_10k -ifp rad64day3_R10k -method dise -metric tanimoto -n 500 -d 0.4 -o dise500.out”.
