# Supplementary material for: How Size Matters: Diversity for Fragment Library Design
Source: Molecules. 2019 Aug 5;24(15):2838. doi: 10.3390/molecules24152838 (PMC6696339; doi:10.3390/molecules24152838)
Supplement: Supplementary file 1 [file molecules-24-02838-s001.zip › Table S2.docx]

**Table S2.** Numerical values of diversity metrics calculated for all selected libraries.

|  | **Selected size** | **Similarity^1^** | | | **Richness^1^** | | | **True diversity^1^** | | |
| --- | --- | --- | --- | --- | --- | --- | --- | --- | --- | --- |
|  |  | Diversity-based | Random average^2^ | Random standard deviation^3^ | Diversity-based | Random average^2^ | Random standard deviation^3^ | Diversity-based | Random average^2^ | Random standard deviation^3^ |
| Regular fragments | 100 | 0.122265 | 0.201464 | 0.005482 | 2,887.0 | 1,984.0 | 20.8 | 1,608.44 | 900.95 | 10.07 |
|  | 200 | 0.134625 | 0.225916 | 0.007297 | 5,422.0 | 3,581.7 | 60.5 | 2,466.10 | 1,279.63 | 26.17 |
|  | 500 | 0.154578 | 0.258419 | 0.003808 | 12,148.0 | 7,842.3 | 34.1 | 3,993.04 | 1,894.26 | 10.98 |
|  | 1,000 | 0.174402 | 0.285495 | 0.001213 | 21,673.0 | 13,764.3 | 198.9 | 5,246.50 | 2,400.40 | 45.61 |
|  | 2,000 | 0.196343 | 0.321847 | 0.001272 | 38,363.0 | 23,807.7 | 76.3 | 6,611.95 | 2,957.03 | 17.29 |
|  | 5,000 | 0.233210 | 0.370939 | 0.003388 | 78,573.0 | 49,109.3 | 520.0 | 8,107.79 | 3,807.88 | 52.58 |
|  | 10,000 | 0.268482 | 0.413619 | 0.001703 | 130,584.0 | 83,253.7 | 514.6 | 8,834.95 | 4,463.67 | 55.68 |
|  | 20,000 | 0.313856 | 0.461026 | 0.000562 | 209,327.0 | 137,657.7 | 493.3 | 9,087.67 | 5,051.36 | 26.63 |
|  | 50,000 | 0.395056 | 0.525136 | 0.000379 | 358,545.0 | 260,768.3 | 212.7 | 8,576.17 | 5,797.96 | 10.54 |
|  | 100,000 | 0.478154 | 0.574659 | 0.000529 | 503,611.0 | 409,410.0 | 1,137.9 | 7,896.44 | 6,241.94 | 18.94 |
| Fluorinated fragments | 100 | 0.149612 | 0.252146 | 0.017761 | 2,965.0 | 1,891.3 | 70.2 | 1,449.76 | 767.69 | 22.75 |
|  | 200 | 0.170762 | 0.269301 | 0.017459 | 5,327.0 | 3,420.7 | 110.1 | 2,003.75 | 1,058.97 | 54.73 |
|  | 500 | 0.196946 | 0.323173 | 0.001232 | 11,251.0 | 7,089.3 | 46.2 | 2,830.74 | 1,446.08 | 18.91 |
|  | 1,000 | 0.222439 | 0.354873 | 0.004163 | 19,173.0 | 12,366.3 | 48.2 | 3,455.38 | 1,826.25 | 22.53 |
|  | 2,000 | 0.256078 | 0.399990 | 0.002119 | 32,205.0 | 21,121.7 | 77.7 | 3,982.73 | 2,176.42 | 16.95 |
|  | 5,000 | 0.313105 | 0.460942 | 0.001571 | 60,407.0 | 41,785.0 | 203.8 | 4,425.32 | 2,708.11 | 36.95 |
|  | 10,000 | 0.374811 | 0.509891 | 0.001579 | 92,096.0 | 67,448.0 | 321.1 | 4,452.28 | 3,025.40 | 18.07 |
|  | 20,000 | 0.456521 | 0.559276 | 0.000733 | 130,417.0 | 106,525.7 | 170.9 | 4,198.97 | 3,325.51 | 8.28 |

^1^ Calculation methods for these three diversity metrics were detailed in the main text.

^2^ Average values of triplicates.

^3^ Standard deviations were calculated by the STDEV function of Microsoft Excel.
