# Supplementary figures and images for: How Size Matters: Diversity for Fragment Library Design
Source: Molecules. 2019 Aug 5;24(15):2838. doi: 10.3390/molecules24152838 (PMC6696339; doi:10.3390/molecules24152838)

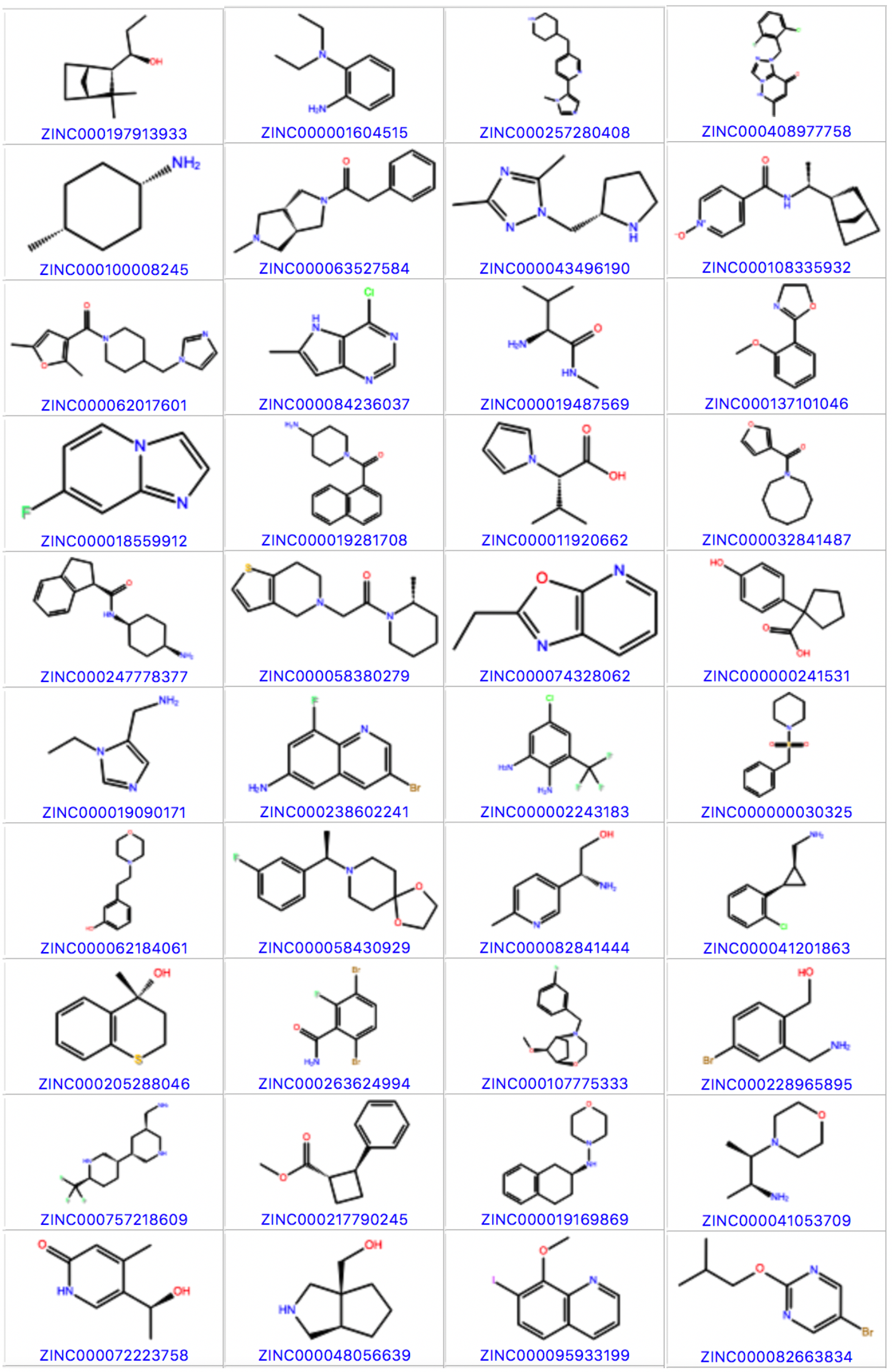

Supplement: Supplementary file 1 [file molecules-24-02838-s001.zip › Fig_S1.tif]
